# Association of maxillary dental developmental abnormality with precocious puberty: a case-control study

**DOI:** 10.1186/s40902-020-00274-3

**Published:** 2020-08-26

**Authors:** Yesel Kim, Nam-Ki Lee, Jae Hyun Kim, Jeong-Kui Ku, Bu-Kyu Lee, Hoi-In Jung, Sun-Kyu Choi

**Affiliations:** 1 Department of Preventive Dentistry and Public Oral Health, Yonsei University, Seoul, Korea; 2 Department of Orthodontics, Section of Dentistry, Seoul National University Bundang Hospital, Seongnam-si, Korea; 3 Department of Pediatrics, Seoul National University Bundang Hospital, Seongnam-si, Korea; 4 Department of Oral and Maxillofacial Surgery, Section of Dentistry, Armed Forces Medical Command, Armed Forces Capital Dental Hospital, Seongnam-si, 13634 Korea; 5 Department of Oral and Maxillofacial Surgery, Seoul Asan Medical Center, Seoul, Korea; 6 Department of Biostatistics, Korea University College of Medicine, Seoul, Korea

**Keywords:** Dental developmental abnormality, Gonadotropin-releasing hormone, Mesiodens, Precocious puberty, Supernumerary tooth

## Abstract

**Background:**

Dental studies of precocious puberty have focused on examination of jaw and dentition growth. The aim of the study was to analyze the relationship between precocious puberty and maxillary dental developmental abnormalities (DDAs).

**Methods:**

This retrospective study was conducted on the Korean patients in whom dental panoramic and hand-wrist radiographs had been taken before they were 15 years of age. The maxillary DDAs were assessed as mesiodens, congenital missing teeth, peg-shape lateral incisors, or impacted teeth. The chronological ages of the control group members were within the normal range of the hand-wrist bone age. Others with a peak luteinizing hormone of ≥ 5 and < 5 IU/L were allocated to central precocious puberty (CPP) and peripheral precocious puberty (PPP), respectively.

**Results:**

Of the enrolled 270 patients, 195, 52, and 23 were allocated to the control, CPP, and PPP groups, respectively. The maxillary DDAs were significantly more prevalent in the CPP group than in the other groups. Among those with maxillary DDA, the mesiodens predominated. Age- and sex-adjusted multivariate analysis revealed maxillary DDA (odds ratio, 3.36; 95% CI, 1.60-7.05) and especially mesiodens (odds ratio, 5.52; CI, 2.29-13.28) to be significantly associated with CPP.

**Conclusions:**

Maxillary DDAs were significantly more prevalent in the CPP group than in the PPP or control groups. Among the many types of maxillary DDAs, mesiodens was significantly associated with CPP and may be considered a predictor of the development of CPP.

## Background

Precocious puberty (PP) has recently become a topic of social focus. PP can be identified by signs of pubertal development in girls aged < 8 and in boys aged < 9 [1]. When the bone age—as determined using hand-wrist radiography by the Tanner-White or Greulich-Pyle atlas method [2]—is advanced compared to the chronological age, PP can be diagnosed differentially as central precocious puberty (CPP) or peripheral precocious puberty (PPP) using a gonadotropin-releasing hormone stimulation test (GnRHST) [3–6].

Treatment of PPP is focused on the originated diseases such as congenital adrenal hyperplasia, McCune-Albright syndrome, severe hypothyroidism, disorders of the adrenal gland, tumors of the ovary or testis, and rare genetic syndromes [4]. On the other hand, the goal of treatment for CPP patients can be considered to match pubertal development with their peers to reduce the psychosocial problems and minimize the loss of growth potential. In patients with CPP, delayed treatment may result in growth loss and socio-psychological problems, such as emotional distress and problem behavior, because hormonally caused behavioral changes (e.g., aggression) may break out earlier in patients with CPP [7, 8]. Therefore, many studies have been conducted to identify the predictive factors of CPP which has been attributed to a dysfunction of the hypothalamic-pituitary-gonadal axis. As a result, endocrine-disrupting BMI [6], chemicals [7], central nervous (CNS) problems, or head trauma [8] have been suggested to be predictors of the future development of CPP.

At the chronologic age of six to seven, before puberty begins, mixed dentition starts as the deciduous teeth which are replaced with permanent teeth. Since the timing of PP diagnosis is an important dental turning point, many researches have been conducted on the relationship between PP and dental development such as tooth eruption, tooth growth, and jaw growth [9–12]. However, the relationship between PP and the dental parameter is controversial because the above parameters vary, even in individuals without PP.

Dental developmental abnormalities (DDAs) are evidenced by an abnormal tooth shape or number such as peg-shaped maxillary lateral incisors (peg-lateralis), congenital missing tooth, impacted maxillary permanent teeth, germinated tooth, fused tooth, twinned tooth, taurodontism, or supernumerary teeth. The DDAs are more common in the maxillae than mandibles [13, 14]. Of these DDAs, supernumerary teeth are usually encountered in the anterior maxillae and are called mesiodens [14]. No association between mesiodens and other DDAs has been reported whereas peg-lateralis, congenital missing lateral incisors, and impacted canines are interrelated [15, 16]. Before the identification of pubertal development, most DDAs can be diagnosed easily using dental radiographs. And the DDAs are recommended to treat approximately before maxillary permanent incisor eruption (5 to 6 years of age) [17].

Both maxillary teeth and the anterior pituitary gland, the latter of which secretes follicle-stimulating hormone (FSH), luteinizing hormone (LH), and growth hormone (GH), are embryologically derived from the oral epithelium. Therefore, maxillary DDA may be embryologically associated with PP. In addition, maxillary DDA may be a valuable predictor of a diagnosis of PP because they can be identified before the onset of pubertal development. The aim of this study was to identify the relationship between maxillary DDA and PP.

## Materials and methods

This case-control study was conducted on patients in whom dental panoramic and hand-wrist radiographs had been taken between March 2008 and May 2018, at the department of pediatrics or dentistry in Seoul National University Bundang Hospital. The inclusion criteria were as follows: (1) age between 3 and 15 years when both dental panoramic and hand-wrist radiographs were taken and a hand-wrist evaluation for bone age using the Greulich-Pyle atlas method [2]. (2) The presence of GnRHST results in a patient with advanced bone age compared to the chronological age. The exclusion criteria were as follows: history of orthodontic treatment, maxillofacial surgery, and the presence of dentofacial-related deformity or syndrome.

The range of bone age was determined as by expert radiologists with the Greulich-Pyle atlas method. The control group consisted of patients in whom their chronological age within the range of bone age. Among other patients with an earlier bone age than the chronological age and breast budding or testicular enlargement, they were classified into the experimental groups (CPP and PPP groups) by GnRHST (described below). Gonadotropin-releasing hormone (GnRH, 100 μg; Relefact; Sanofi-Aventis, Frankfurt, Germany) was injected intravenously after obtaining baseline serum samples. Luteinizing hormone (LH) was measured by blood samples which were collected 30, 45, and 60 min after GnRH administration. The experimental group was divided into a CPP group with a peak LH concentration of ≥ 5 IU/L and a PPP group with a peak LH concentration of < 5 IU/L [4].

**Fig. 1:**
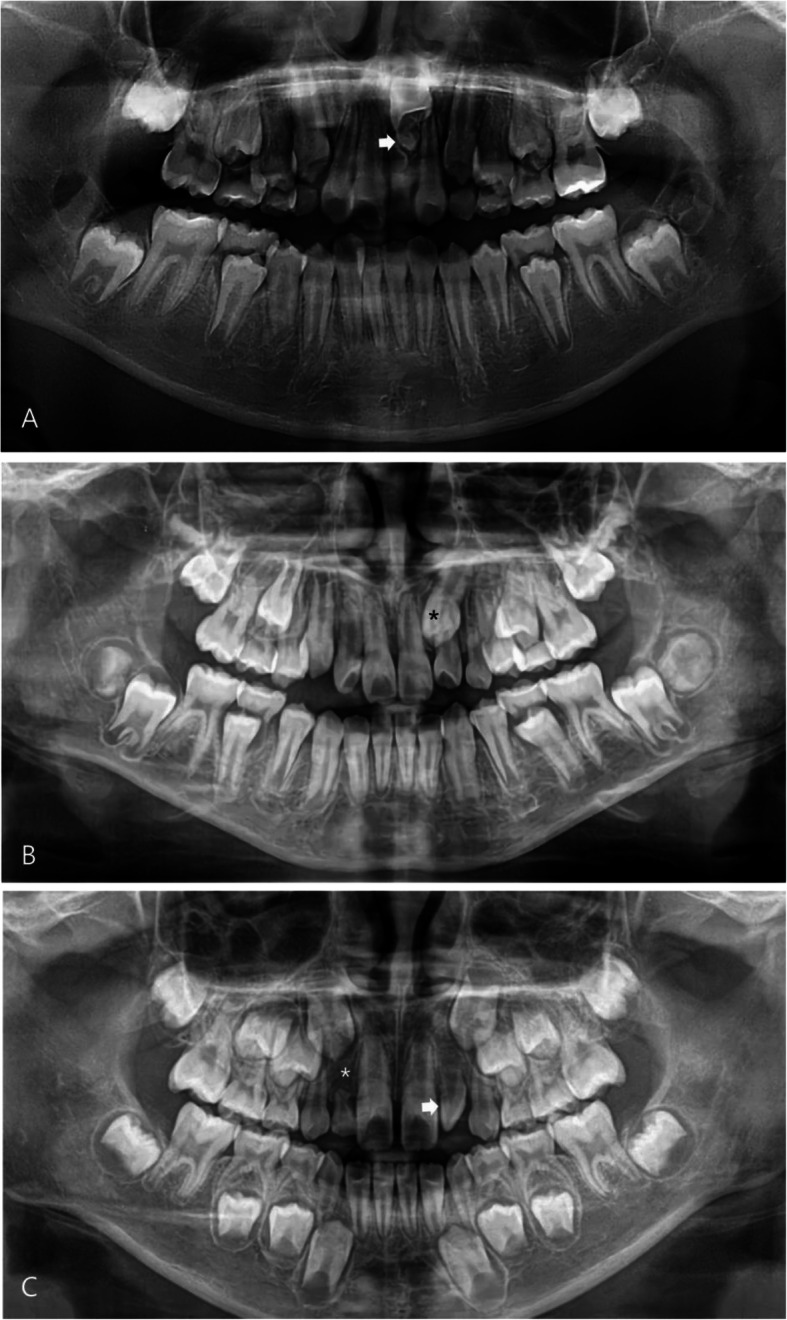
Maxillary dental developmental abnormality. **a** Mesiodens (arrow). **b** Impacted maxillary canine (asterisk). **c** Congenital missing of the lateral incisor (asterisk) and peg lateralis (arrow)

Because the patients independently visited the department of dentistry and pediatrics, the pediatric evaluation age was defined as chronological age at the time when the hand-wrist radiograph was first examined, and the dental evaluation age was separately defined at the time when the first dental panoramic radiograph was taken. Statistical analysis was based on the dental evaluation age. In the dental panoramic radiographs, the maxillary DDAs were classified into mesiodens and the others, including impacted maxillary permanent teeth, congenital missing teeth, and peg lateralis, by an expert oral and maxillofacial surgeon (Fig. 1).

### Statistical methods

The control, CPP, and PPP groups were determined using a chi-square or Fisher’s exact test for the categorical variables or ANOVA for the continuous variables. Post hoc analysis was performed by Bonferroni correction. Statistical significance among the groups was evaluated according to the subtypes of maxillary DDAs (total maxillary DDAs, mesiodens, and other maxillary DDAs except for mesiodens). One to one propensity score matching to adjust for age and sex ratio was applied to the dataset of the control and CPP group. The significance of maxillary DDAs in predicting the development of the PP response was compared using both univariate and multivariate logistic regression analysis to adjust for age and sex ratio. The univariate and multivariate odds ratio with their 95% confidence intervals were calculated for the subtypes of maxillary DDAs. Two-sided *p* values of < 0.05 were considered significant. The analysis was performed using SAS version 9.4 (SAS Institute, Cary, NC) and R 3.5. 1 (Vienna, Austria; http://www.R-project.org/).

### Ethics statement

The study was reviewed and approved by the Institutional Review Board at Seoul National University Bundang Hospital (No. B-1904/535-106). It was granted an exemption of the informed consent due to the retrospective nature of this study.

## Results

**Fig. 2:**
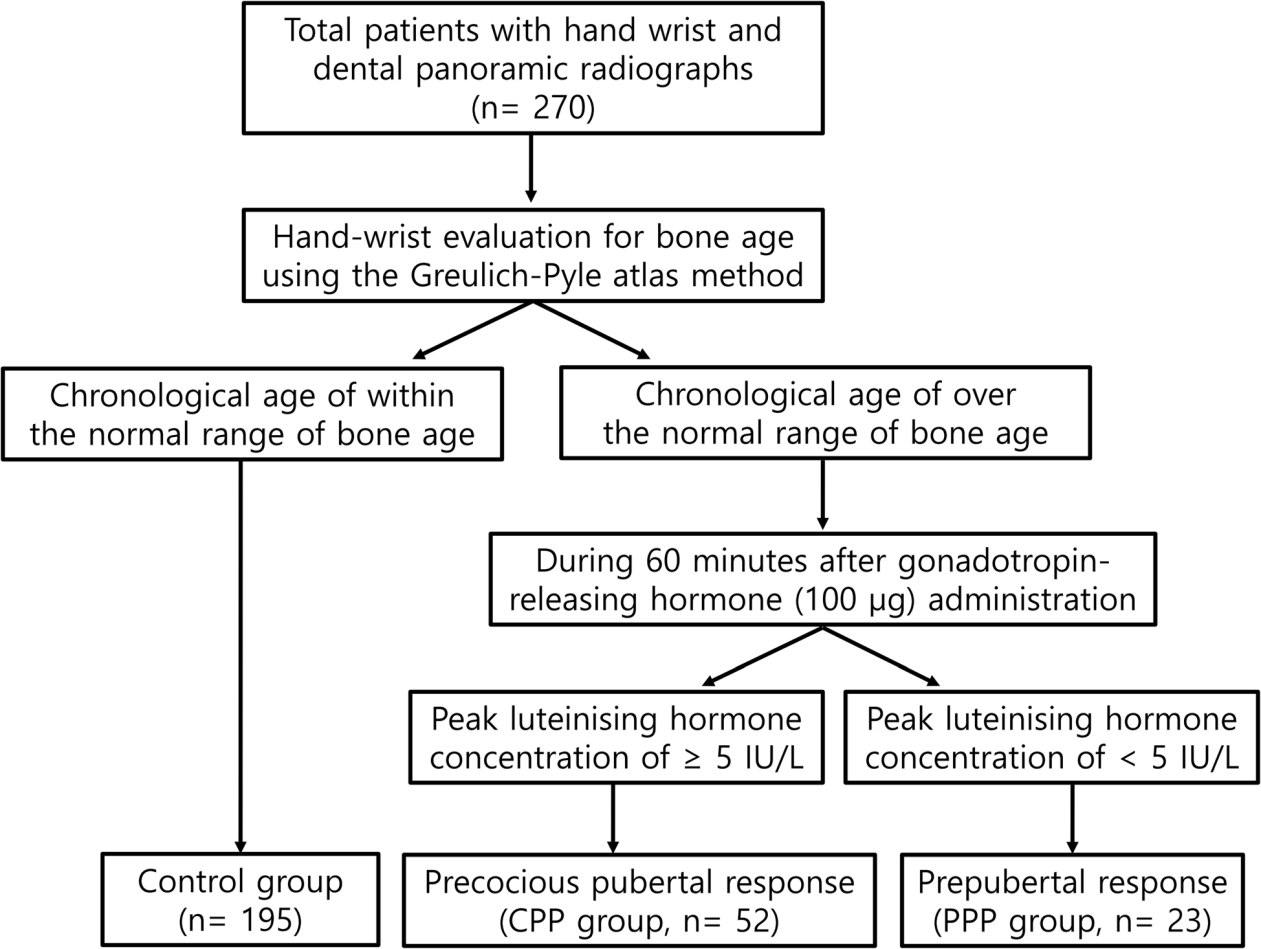
Flow diagram of patient classification

Two hundred and seventy patients (12.3 ± 4.4 years) were enrolled in this study; 195, 52, and 23 were allocated to the control, CPP, and PPP groups, respectively (Fig. 2). The pediatric evaluations were performed earlier on average than the dental evaluations in all groups. CPP and PPP groups revealed significant intergroup differences in sex and age compared with control group (Table 1).

**Table 1:** Demographic characteristics of the subjects and prevalence of maxillary dental developmental abnormalities in the three study groups

No.	Control group *n* = 95	CPP group *n* = 52	PPP group *n* = 23	*P*
Female (%)	109 (55.9 %)	47 (90.38 %)	23 (100 %)	<.001^a^ (Control vs CPP, PPP)
Age (year)				
Pediatric evaluation	10.5 ± 2.9	8.3 ± 0.5	8.0 ± 1.1	<.001^b^ (Control vs CPP) .023^b^ (Control vs PPP)
Dental evaluation	12.0 ± 4.4	8.9 ± 2.3	9.6 ± 3.3	<.001^b^ (Control vs CPP, PPP)
Prevalence				
Maxillary DDAs	43 (22.05 %)	26 (50.00 %)	5 (21.74 %)	<.001^a^ (Control vs CPP) 1.000^a^ (Control vs PPP) <.029^a^ (CPP vs PPP)
Mesiodens	26 (13.33 %)	22 (42.31 %)	5 (21.74 %)	<.001^a^ (Control vs CPP) .732^a^ (Control vs PPP) <.049^a^ (CPP vs CPP)
Except mesiodens	17 (8.71 %)	4 (7.69 %)	0	0.317^**c**^

Abbreviations: *CPP* central precocious puberty, *DDAs* dental developmental abnormalities, *PPP* peripheral precocious puberty

^a^Post hoc analysis after chi-square test

^b^Post hoc analysis after ANOVA

^c^Fisher exact test compared with control group

The prevalence of maxillary DDA was significantly higher in the CPP group (50.00%) than in the control (22.05%) and PPP (21.74%) groups (*P* < 0.05). Mesiodens was more prevalent in the CPP group (42.31%) than in the control (13.33%) and PPP (21.74%) groups (*P* < 0.05). But no intergroup difference was observed between the prevalence of the other maxillary DDAs, except for the mesiodens, in the PPP (0.00%), CPP (7.69%), and control (8.71%) groups (Table 1).

One-to-one propensity score matching was used to adjust for age and sex ratio, which differed in the control and CPP groups. The prevalence of maxillary DDAs were significantly different in the CPP (50.00%) and control (19.23%) groups (*P* < 0.001). The prevalence of mesiodens was significantly higher in the CPP group (42.31%) than in the control group (9.53%) (*P* < 0.001). On the other hand, the prevalence of the other maxillary DDAs, except for mesiodens, was similar in the control (11.54%) and CPP (7.69%) groups (Table 2).

**Table 2:** Prevalence of maxillary dental developmental abnormalities in the control group and precocious pubertal response group after 1:1 propensity score matching (*n* = 52)

	No. (%)		*P*
	Control group	CPP group	
Maxillary DDAs	10 (19.23 %)	26 (50.00 %)	0.001^**a**^
Mesiodens	4 (7.69 %)	22 (45.31 %)	< 0.001^**a**^
Except mesiodens	6 (11.54 %)	4 (15.38 %)	1^**b**^

Abbreviations: *CPP* central precocious puberty, *DDAs* dental developmental abnormalities

^a^Chi-square test

^b^Fisher exact test

Univariate analysis and age- and sex-adjusted multivariate analysis revealed maxillary DDAs (odds ratio, 3.85; 95% confidence interval, 1.92-6.92 and 3.36; 1.60–7.05, respectively), especially mesiodens (odds ratio, 4.98; 95% confidence interval, 2.46–10.06 and 5.52; 2.29–13.28, respectively), to be associated with the precocious puberty response (Table 3).

**Table 3:** Predictors of the precocious pubertal response, including the univariate and adjusted odd ratio and 95% confidence limits according to maxillary dental developmental abnormalities

Control group vs CPP group	Odd ratio (95 % CI)	
	Unadjusted^**a**^	Adjusted^**b**^
Maxillary DDAs	3.85 (1.92-6.92)	3.36 (1.60-7.05)
Mesiodens	4.98 (2.46-10.06)	5.52 (2.29-13.28)
Except mesiodens	1.39 (0.43-4.44)	

Abbreviations: *CPP* central precocious puberty, *CI* confidence interval, *DDAs* dental developmental abnormalities

^a^Univariate logistic regression

^b^Adjustment of age and sex

## Discussion

In this study, the relationship between maxillary DDAs and PP was firstly analyzed retrospectively. In general, it is important that children with CPP be identified from normal and PPP early because delayed diagnosis and the treatment of CPP leads to a loss of growth potential and psycho-social problems [1, 18]. Many studies have been conducted to identify the screening or predictive factors of CPP. The relationship remains controversial between the CPP and dental factors such as dental maturity [10, 19], dental age [11], malocclusion [12], and mandibular growth pattern [10]. At time of PP onset, many children with mixed-dentition visited dental clinics to have their dental development evaluated. For this dental examination, the patients are radiographed to examine the eruption of permanent teeth or identify the DDAs including supernumerary, impacted, and missing teeth. DDAs can be diagnosed clearly and efficiently using radiographs by the abnormality of shape or number of tooth [17]. The supernumerary tooth—mesiodens—occurs at the time of maxillary permanent tooth germ formation [20]. The enamel portion of the maxillary permanent anterior teeth is formed between 3 to 4 years of age, and the age of eruption of these teeth is commonly between 6 to 7 years of age. Therefore, DDA may be a predictive factor in the early diagnosis of CPP because DDA can be identified before the onset of signs of pubertal development.

Embryologically, the pituitary gland is an important structure for the migration of neural crest cells involved in oral formation [21]. The anterior pituitary gland has the same origin as oral neural crest cells. And the posterior pituitary gland has the same mesenchymal origin as the maxillofacial region [22]. The sella turcica forms a bony seat for the pituitary gland. Therefore, a sella turcica deformity has been reported to be associated with tooth developmental disorders including mesiodens [23–26]. In addition, an abnormality of the sella turcica has been proven to be related to tooth eruption by an analysis of the eruption timing and eruption disorders of the maxillary teeth according to the nerve distribution [22]. On the other hand, the effect of hormones remains controversial on tooth maturity or jaw growth. Some studies have reported a relationship between the hormones secreted by the pituitary gland and the development of dentition or jaw growth. Kjellberg et al. reported that patients with a GH deficiency exhibited delayed tooth eruption [27], and Cantu et al. reported that GH-deficiency patients showed a delayed bone age but no delay in dental age on the radiographs [28]. Others have reported that sexual maturity is not related to dental maturity [29], and a GH treatment has little effect on tooth development [30, 31].

GnRHST is a test method commonly used to diagnose CPP [5]. The test is used to evaluate the activity of the hypothalamic-pituitary axis by measuring the amount of releasing concentration of LH and FSH. GnRHST is considered invasive given the patients’ ages because the test requires two to three consecutive blood tests separated by intervals of 15–30 min [5]. For this reason, a screening examination is performed by evaluating the bone age using a hand-wrist radiograph [19]. In the present study, the control group included patients whose bone age (as determined using the Greulich-Pyle atlas method) did not exceed the chronological age. Among the patients with an advanced bone age than chronological age, the CPP and PPP groups were classified based on the peak LH above 5 lU/L [4].

The CPP group had a significantly higher prevalence of maxillary DDAs than the control or PPP groups. Statistical analysis was performed between the CPP and control groups with an adjustment for sex and age because the proportion of girls was higher in the CPP group at 90.38%, but no sex difference was observed in the control group at 55.90%. The prevalence of maxillary DDAs was higher in the CPP group; regression analysis showed that the odds of maxillary DDAs in this group were 3.36 times higher than in the control group (confidence interval; CI, 1.60-7.05). In addition, mesiodens was remarkably prevalent in the CPP group, and the odds ratio of mesiodens was 4.98 times higher (CI, 2.46-10.06) in the CPP group. After sex and age-adjusted regression analysis, the odds ratio increased to 5.52 times higher (CI, 2.29–13.28) for mesiodens in the CPP group than the control group (Table 3). As a consequence, mesiodens could be considered a strong predictor of the development of CPP. As the CNS problem becomes a well-known risk factor for PP, CPP boys were frequently found among the CNS problem patients [8]. Similarly, mesiodens could be used to predict CPP in boys who may not be diagnosed. Furthermore, CPP might be determined at the gestation period because mesiodens is developed around at the 16th week of gestation [20].

Early diagnosis and treatment of DDA patients are also important for increasing spontaneous tooth eruption and reducing the need for additional orthodontic or surgical intervention as well as the development of psychosocial problems [17]. In this study, however, the average dental evaluation age of the patients was slightly later than those of the pediatric evaluation age. The authors indicate that they might miss the opportunity for the early diagnosis of maxillary DDAs because no associations between the DDAs and PP have been established. Therefore, patients diagnosed with CPP should be referred for a dental examination. Furthermore, it should be noted that maxillary DDA patients were at high risk of developing CPP. Thus, they need to be referred for a pediatric examination.

On the other hand, DDA has a prevalence of 5-19% in mixed-dentition juveniles. In DDA, the prevalence of supernumerary teeth is generally approximately 3% [17]. Compared to previous studies, the results of this study showed a high prevalence of DDA in all three study groups, which can be attributed to the retrospective single institutional study design. Because not every patient with mesiodens was simultaneously tested for bone age and precocious puberty, it could be possible that the mesiodens patients were not evenly distributed among the groups. In addition, the risk of PP in boys with maxillary DDA could not be evaluated because of the limited number of samples. This study had some limitations, such as small sample size, heterogeneous sex distribution, and retrospective cohort study. Further large-scale or prospective multicenter studies of the association between CPP and maxillary DDAs, particularly mesiodens, will be needed.

## Conclusions

Maxillary DDA was associated in the CPP group compared with the PPP or control groups. In particular, mesiodens was associated significantly with the PP response, and could be considered a predictor of CPP development. A patient diagnosed with CPP needs to be referred for a dental examination, and those identified with mesiodens should be referred for a pediatric examination of CPP.

## Data Availability

The datasets used during the current study are available from the corresponding author on reasonable request.

## Abbreviations

CPP: Central precocious puberty
CI: Confidence interval
DDAs: Dental developmental abnormalities
PP: Precocious puberty
PPP: Peripheral precocious puberty
